# Novel hybrid medical curriculum in a developing country: Experience of medical students

**DOI:** 10.15694/mep.2020.000242.1

**Published:** 2020-10-29

**Authors:** Wafaa Mowlabaccus, Zuhair Hussain Mohamed, Adnaan Mowlabaccus

**Affiliations:** 1 University of Mauritius

**Keywords:** medical curriculum, personal views, medical students

## Abstract

This article was migrated. The article was marked as recommended.

The aim of this article is to share the experience of final year medical students on being in the first cohorts of a new hybrid medical curriculum. It discusses the opinions and views on the structure of the curriculum as well as pointing out some of the challenges faced by medical students.

## Introduction

While we read the paper on “Innovative Curriculum prepares medical students for a lifetime of learning and patient care” by Bruce Dubin ( Dubin, 2016), we reflect on the medical curriculum we have been exposed to and the challenges we faced as students. The University of Mauritius (UOM) in collaboration with the Ministry of Health and Wellness, and the University of Geneva (UNIGE) developed an English based undergraduate medical program which started in 2013. It consists of 3 years full-time Bachelor of Medicine followed by 3 years full-time Master of Medicine which then leads to the award of Doctor of Medicine (MD) degree. The amalgam of the ***CanMed 2005 framework*** ( Frank *et al.*, 2005), the British ***‘Curriculum for the foundation years in postgraduate education and training’*** ( Umscheid, Margolis and Grossman, 2011) and the ***Swiss Catalogue of learning objectives for undergraduate Medical training***- June 2008 ( Working Group under a Mandate of the Joint Commission of the Swiss Medical Schools, 2008) formed the core learning objectives of the novel MD program in Mauritius.

During our first year we had mainly lecture based classes which covered disciplines such as chemistry, physics, anatomy, genetics, physiology and biochemistry. For our 2 ^nd^ and 3 ^rd^ years we had Problem Based Learning (PBL) alongside with lectures, practical and clinical skills. 4 ^th^ and 5 ^th^ year consisted of clinical posting, Case Based Learning (CBL) and a project. Our final year consisted exclusively of clinical postings for a duration of 10 months followed by the final exams.

## Day to day challenges encountered

Being in the first cohorts of a new medical curriculum was a challenging and unique experience for us. Since the beginning of the course, we came across several technical hitches. For instance, we still remember the days when we had to look for classrooms in the university to have lectures and liaise with our lecturers on our own since we had no designated program assistant. Since our university was still carrying out recruitment of full time and part-time lecturers, it was not uncommon for us to have one lecturer carrying out several lectures for different disciplines in our first year. Moreover, having no seniors to ask advice from, we were prompted to become autonomous and proactive since day one. We had to devise our own means and strategies to cope with the overwhelming medical curriculum.

Our exams being mostly multiple choice questions with OSCE (Objective Structured Clinical examination) as from the 3 ^rd^ year were carried out by the university and vetted by external examiners. While we had no past exams papers to practice, we had to seek help from online question banks for other board exams papers such as USMLE (United States Medical License Examination) **,** PLAB (Professional and Linguistic Assessment Board) and MRCP (Membership of the Royal Colleges of Physicians) to practice questions with the hope of obtaining some experience on how to answer multiple choice questions and what to expect from OSCE questions.

## Problem based learning (PBL)

Problem based learning (PBL) which took place in our 2nd and 3rd year was a new concept for both the tutors and medical students. We really indulged and enjoyed ourselves as the problem scenarios consisted of real-life cases which were interesting and triggered active learning. We also learnt important skills like working in collaboration with our peers as well as becoming self-directed learners. While we were, at first, quite lost with the new PBL method, the tutors made us feel both at ease and helped us to appreciate this new learning activity. Though we felt that most of the tutors carried out their role as coaches, guided us through group discussion and ensured we understood important concepts, we also perceived that some of them lacked training and were under experienced and they had a tendency to lead the group, thus dampening the critical thinking of students. As with most of group works, we also faced challenges concerning the group dynamics in some PBL. A lack of interest from peers, low active participation and repeated absences from certain groupmates led to underachievement of the objectives set for the PBL sessions. Moreover, since there was a shortage of staff and part-time lectures had varying timetables, we were sometimes unable to have PBL sessions and lectures based on the PBL concomitantly. This had a negative impact on our study since we felt that self-study was not sufficient to grasp certain important concepts. For example, in our cardiac module, we did not have a lecture on interpretation of ECG in parallel with the PBL sessions, which made it hard to understand some of the PBL cases. Finally, being buried in multiple resources for self-learning and our objectives sometimes being too vague, often steered us to go into too much details that was beyond the scope of the PBL.

## Clinical postings - the good, the bad and the ugly

Most of our clinical postings were carried out at one major hospital which was undergoing conversion into a teaching hospital. We were exposed to real patients having medical problems similar to those in our PBL and Case Based Learning (CBL) scenarios and thus were given the opportunity to link previous concepts learned and to apply them to the real cases in the wards. However, lack of communication between the university and the teaching hospital led to most of the physicians being unaware of our learning objectives which then resulted in medical students to be left un catered for. We did not have any structured bedside teaching during our first months of rotation and since we had been only practicing on ourselves and standardized patients it was a steep learning curve to apply the same examination techniques on real patients. Furthermore, as medical students, we were expected to integrate ourselves into the system without asking too many questions. This pushed us to become autonomous, inquisitive and resilient. We had the opportunity to meet patients by ourselves, take relevant history and examine them and we asked for help from the nurses when we had to attempt any technical skills. Our learning depended on how devoted and enthusiastic we were as medical students since if we did not ask questions and try to integrate in the system, there was not much scope to learn.

## Conclusion

In conclusion, it is without a doubt that being in the first cohorts of a novel medical curriculum was thought-provoking, stimulating and challenging. We believe that all the hurdles we faced along the way have shaped us into becoming self -learned individuals who are pro-active, resilient and autonomous. After six long years, we hope to integrate the health care system as foundation doctors who have been holistically trained for the near future.

## Take Home Messages

• Being a medical student in the first cohorts of a new hybrid medical curriculum is a unique and challenging experience.
• Being proactive and resilient can help medical students to pave their way through medical school.
• Despite many obstacles that may present along the way, it is possible to fight our way to achieve a goal.

## Notes On Contributors

**Wafaa Mowlabaccus** is a final medical student at the University of Mauritius currently studying the Doctor of Medicine programme. ORCID ID: https://orcid.org/0000-0003-3984-4203

**Zuhair Hussain Mohamed** is a medical graduate from the University of Mauritius. ORCID ID: https://orcid.org/0000-0003-3793-0933

**Adnaan Mowlabaccus** is a medical graduate from the University of Mauritius.
