# Supplementary material for: Novel hybrid medical curriculum in a developing country: Experience of medical students
Source: MedEdPublish (2016). 2020 Oct 29;9:242. [Version 1] doi: 10.15694/mep.2020.000242.1 (PMC10697569; doi:10.15694/mep.2020.000242.1)
Supplement: Peer review history

# Reviewer report: Reviewer response for version 1

**DOI:** 10.21956/mep.20181.r31207

**Published:** 2020-12-24

**Authors:** Ken Masters

**Affiliations:** 1 Sultan Qaboos University

This review has been migrated. The reviewer awarded 3 stars out of 5 An interesting students’ personal viewpoint article about a novel hybrid medical curriculum in a developing country.Being the first cohort in a new curriculum is always challenging, and the paper outlines the typical range of difficulties that are bound to be experienced. A new curriculum introduces not only new material, but also new methods – in this paper, PBL. The introduction of PBL is always difficult, and requires a great deal of support – for both students and faculty – if it is to be performed correctly and with the intended benefits.The advantages, however, appear to have been clear to the students, and, from the paper, it appears that they utilised these. Usually, where there is this novelty and uncertainty, there are opportunities for self-realisation that would otherwise not present themselves (and which will probably be curtailed in the future, as the process becomes more formalised). Recognising and grasping these opportunities is key to drawing full advantage from the uncertain situation.It would have been more useful if more detail had been given; In spite of that, although much of what is described in the paper is not generalisable, it might be inspiring to other students who find themselves as the first cohort in a new curriculum. At the very least, they may take heart from the fact that many students have had to face this challenge, and success comes from dealing with the obstacles and opportunities as positively as possible.

Reviewer Expertise:

NA

No decision status is available

# Reviewer report: Reviewer response for version 1

**DOI:** 10.21956/mep.20181.r31208

**Published:** 2020-10-30

**Authors:** P Ravi Shankar

**Affiliations:** 1 American International Medical University

This review has been migrated. The reviewer awarded 4 stars out of 5 I enjoyed reading this article. The first cohort of students at a new institution often faces several challenges. The authors have described their curriculum and the challenges they faced very well. In an earlier paper they had also described the challenges they faced with the rapid transition to online learning during the COVID-19 pandemic. A chronological timeline of the evolution of the new medical school will be of interest. When did the first batch of students graduate? How are students selected for the program and what is the batch size? I am happy to note that all the authors are either medical students or recent graduates.

Reviewer Expertise:

NA

No decision status is available
